# Utility of Serum L-lactate in Identifying Ischemia in Acute Intestinal Obstruction: A Prospective Observational Study

**DOI:** 10.7759/cureus.38443

**Published:** 2023-05-02

**Authors:** Aneena A Moncy, Alfie J Kavalakat, B Vikraman

**Affiliations:** 1 Department of General Surgery, Jubilee Mission Medical College and Research Institute, Thrissur, IND

**Keywords:** large intestinal obstruction, predictor factor, serum lactate, intestinal ischemia, small intestinal obstruction

## Abstract

Introduction

In cases of intestinal obstruction, increasing luminal dilatation compromises bowel wall perfusion, eventually resulting in intestinal ischemia and bowel necrosis in advanced cases. Elevated L-lactate, as a biomarker of ischemia, may indicate the presence of bowel ischemia in cases of obstruction. The objective of this study was to evaluate the value of serum L-lactate measurement in predicting the presence of intraoperatively observed intestinal ischemia in patients with acute intestinal obstruction.

Methods

Patients diagnosed with acute intestinal obstruction were prospectively studied over an 18-month period. Serum L-lactate values were assayed twice: at the time of presentation and following appropriate fluid resuscitation. Receiver operating characteristic (ROC) curve analysis was applied to determine the predictive value of serum L-lactate in detecting intestinal ischemia.

Results

One hundred forty-four cases of intestinal obstruction were included in this study, of which 91 underwent operative intervention. Intestinal ischemia was identified in 52 cases and categorized intra-operatively as reversible (n = 33) and irreversible (n = 19). ROC analysis showed a good predictive value of serum L-lactate after fluid resuscitation for irreversible intestinal ischemia (area under the curve (AUC) = 0.884, 95% confidence interval (CI), 0.812-0.956). An L-lactate cut-off of 19.1 mg/dL following fluid resuscitation was determined to have a sensitivity of 89.5%, a specificity of 72.9%, a positive predictive value of 46.6%, and a negative predictive value of 96.3% for gangrenous bowel.

Conclusion

Serum L-lactate is a good predictive tool for identifying intestinal ischemia during the management of intestinal obstruction. Serum L-lactate after resuscitation showed better predictive value for ischemic bowel.

## Introduction

Intestinal obstruction, a common emergency in surgical practice, is complicated by the development of bowel ischemia and necrosis resulting from progressive luminal distension, an increase in intramural tension, and compromised bowel wall perfusion [1-3]. Delayed intervention in cases of suspected ischemia, closed-loop obstruction, and acutely incarcerated or strangulated hernias contributes significantly to patient morbidity and mortality [4,5].

Identifying intestinal ischemia in the early reversible stages, when the bowel can still be salvaged, is important in intestinal obstruction in order to reduce operative morbidity. A national observational study in the United States [6] showed that partial colectomy, small-bowel resection, and lysis of peritoneal adhesions ranked among the top five most commonly performed emergency general surgical procedures. The same study reported mortality rates of 5.3-6.5% associated with emergency resection of the bowel as opposed to 1.6% in cases requiring emergency adhesiolysis.

Though classic signs of vascular compromise include continuous abdominal pain, fever, tachycardia, and signs of peritoneal irritation, these fail to aid in the recognition of ischemic bowel in the early stages [7]. Early and reliable diagnosis is important, and there have been several studies evaluating multiple biomarkers as predictors of bowel ischemia [8-10].

L-lactate is an enantiomer of 2-hydroxypropanoate that occurs naturally in the human body as the end product of anaerobic glycolysis. During conditions of ischemic hypoxia, such as bowel ischemia, oxygen-deprived cells will adopt anaerobic glycolysis, and the level of serum lactate will rise [8,11]. Studies have suggested its potential as a biomarker of intestinal ischemia [12,13], and the World Society of Emergency Surgery (WSES) guidelines for the management of acute mesenteric ischemia state that while no laboratory parameters are sufficiently accurate to conclusively identify ischemic bowel, elevated L-lactate may assist [14]. This study aimed to evaluate the utility of serum L-lactate as a predictor of bowel ischemia in patients with intestinal obstruction.

## Materials and methods

Patients admitted with acute intestinal obstruction were prospectively identified from February 2021 to September 2022 in the General Surgery department of Jubilee Mission Medical College and Research Institute, a single tertiary hospital in Thrissur, India. The inclusion criteria were intestinal obstruction, irrespective of site and cause of obstruction; the diagnosis was made on the basis of standard clinical signs (abdominal pain and distension, nausea or vomiting, no passage of flatus and/or stools) and radiological signs (multiple air-fluid levels on erect X-ray, dilated bowel loops with a transition point on CT of the abdomen). Exclusion criteria were early postoperative obstruction (< two weeks from the index procedure), age younger than 18 years, presence of pneumoperitoneum, and patients with concurrent diabetic ketoacidosis, documented liver cirrhosis, or chronic renal disease on dialysis. One hundred and forty-four patients meeting these criteria were identified and included in this study.

The following items of patient data were collected at presentation: demographic information, patient vitals, laboratory blood test results (white blood cell count, neutrophil-to-lymphocyte ratio, creatinine, lactate), comorbidities (diabetes, hypertension, cardiac disease, etc.), COVID status, history of abdominal surgery, and prior history of intestinal obstruction.

Serum L-lactate values were assessed twice: first, at the time of presentation, and second, following fluid resuscitation of 1 L or as deemed clinically appropriate within a 24-hour period prior to operative intervention. Patients undergoing emergency surgery in less than 24 hours had their second sample of lactate sent prior to the shift to the theater or on the table, i.e., between 4 and 24 hours after starting fluid resuscitation, if considered adequate based on urine output and other resuscitation end-points. Serum L-lactate was assayed using either blood gas estimation or lactate assay, based on the principle of spectrophotometry following the oxidation of lactate by L-lactate oxidase. Reference values of serum L-lactate were 4.5 to 19.8 mg/dL or 0.5 to 2.2 mmol/L.

A subsequent course in the hospital was observed, and the following details were collected: whether the patient underwent non-operative or operative management; duration; and outcome of conservative management. Patients with clinically evident causes of bowel obstruction such as obstructed hernia and large bowel obstruction, as well as cases presenting with features of peritonitis and/or sepsis, were operatively managed.

Cases without such findings were provisionally considered to have an adhesive small bowel obstruction, and if hemodynamically stable, they underwent a trial of non-operative management. Non-operative management was terminated if symptoms worsened or did not resolve in 72 hours. In the event of surgical management, intraoperative findings of the presence of ischemic bowel and cause of obstruction, immediate post-operative outcome and complications, and length of hospital stay.

The primary outcome assessed was the presence of an intraoperatively confirmed irreversible ischemic bowel. Bowel viability was categorized as non-ischemic, reversibly ischemic, and irreversibly ischemic based on the intraoperative assessment of the bowel by the operating surgeon. The definition of bowel status was based on the following descriptions: non-ischemic intestine appears pink and glistening with normal peristalsis and arterial pulsations; reversibly ischemic intestine may appear dusky or congested with a dull sheen and reduced peristalsis and pulsations that significantly improve the relief of obstruction; and irreversibly ischemic intestine appears dark black with no sheen or gangrenous and a permanent absence of normal peristalsis and pulsations.

**Figure 1:**
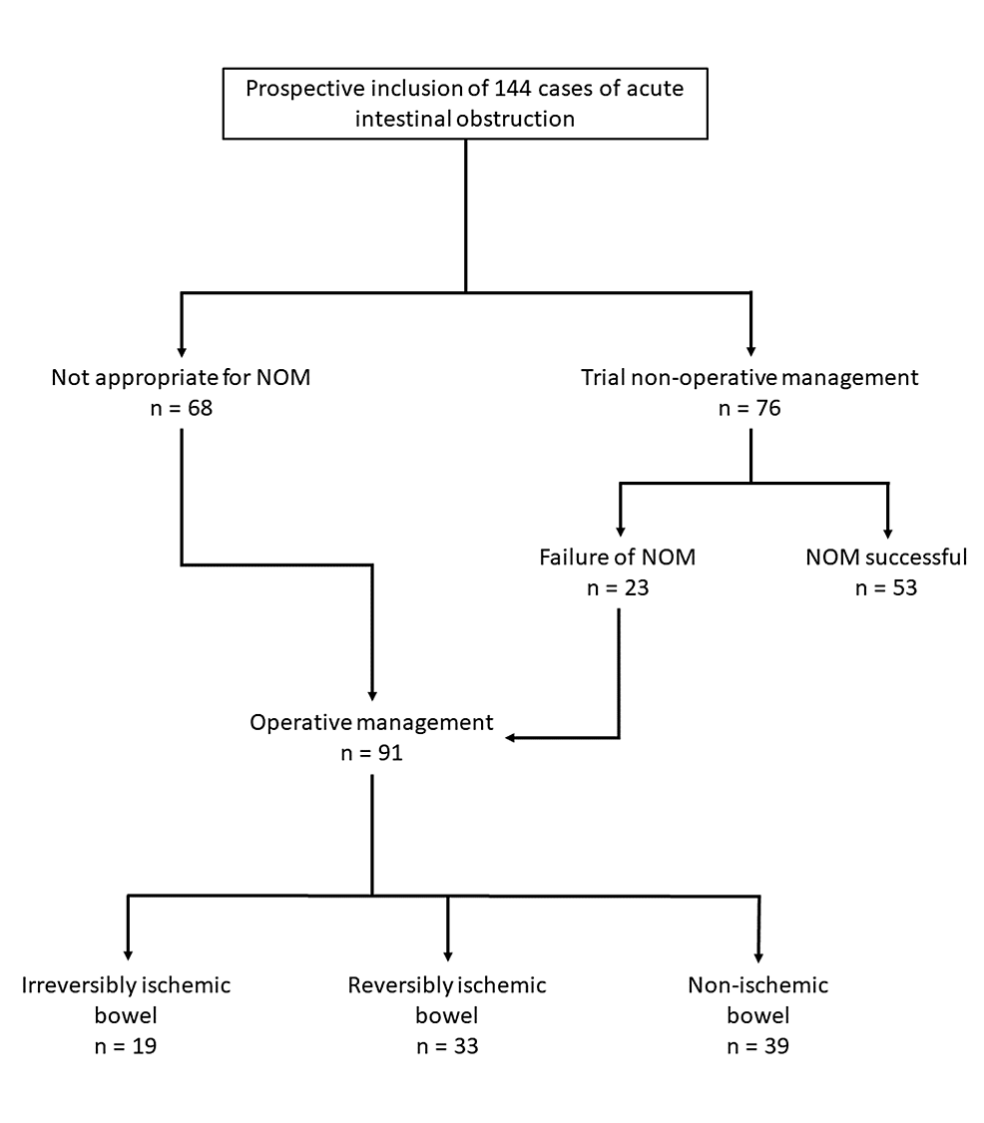
A study of patient disposition NOM: non-operative management

The predictive value of serum L-lactate in anticipating failure of non-operative management in the subset of adhesive small bowel obstruction was studied as a secondary outcome. Patients were also followed up for three months to ascertain the recurrence of obstruction. The study’s patient disposition is shown in Figure 1.

All statistical analysis was performed with IBM Statistical Package for Social Sciences (SPSS) for Windows, version 29.0.0 (IBM Corp., Armonk, New York, USA). Numerical variables (serum L-lactate) were expressed in mean and standard deviation, and categorical variables (presence of intestinal ischemia) were expressed in frequency and percentages. The data were tested for normality prior to the statistical analysis. A p-value of <0.05 was considered significant in all statistical analyses.

Analysis was carried out using the unpaired t-test, Mann-Whitney U, or Kruskal-Wallis H tests for quantitative variables as appropriate, and the chi-square test or Fisher’s exact test for qualitative variables. Receiver operating characteristic (ROC) curve analysis was applied in order to determine the area under the curve (AUC), sensitivity, and specificity of serum L-lactate in detecting intestinal ischemia and evaluate its utility as a predictor of bowel non-viability.

## Results

One hundred and forty-four patients diagnosed with acute intestinal obstruction who met inclusion and exclusion criteria were prospectively identified. The ratio of males to females was 1.5:1, and the median age was 62 years. Serum L-lactate was measured at presentation for all 144 patients and after fluid resuscitation in 142 patients; two patients were operatively managed before repeat sampling.

Fifty-three patients were successfully managed non-operatively, while 91 underwent surgery; 23 of the 91 operated cases required surgery following the failure of non-operative management. Based on operative findings, the level of obstruction was determined to be small bowel (n = 64, 70.3%) and large bowel (n = 27, 29.6%). Causes of obstruction among operated cases were obstructed hernias (n = 28, 30.8%), adhesions (n = 26, 28.6%), malignancy (n = 16, 17.6%), strictures (n = 6, 6.5%), volvulus (n = 4, 4.4%), etc. Intraoperatively, bowel viability was categorized as non-ischemic (n = 39, 42.8%), reversibly ischemic (n = 33, 36.3%), and irreversibly ischemic (n = 19, 20.9%).

Serum L-lactate for predicting intraoperative ischemia

The demographic and clinical data for operatively managed patients are represented in Table 1. The mean observed L-lactate in patients with bowel ischemia (reversible and irreversible) was higher than in patients with non-ischemic bowel (p < 0.001). The neutrophil-to-lymphocyte ratio was also significantly higher in the ischemic bowel group (p = 0.031).

**Table 1:** Demographic variables, comorbidities, and clinical data in patients that underwent operative management based on findings of intra-operative ischemia (n = 91) Continuous variables: mean ± standard deviation (SD) and Kruskal-Wallis test results; categorical variables: n (%) and Pearson's chi-square test results.

Variables	Operative management: non-ischemic bowel (n=39)	Operative management: reversibly ischemic bowel (n=33)	Operative management: irreversibly ischemic bowel (n=19)	p-value
Age, years, mean ± SD	54 ± 19.8	61 ± 14.5	63 ± 16.9	0.120
Gender (male), n (%)	19 (48.7)	23 (69.7)	11 (57.9)	0.198
Diabetes, n (%)	9 (23.1)	7 (21.2)	7 (36.8)	0.420
Hypertension, n (%)	16 (41.0)	9 (27.3)	12 (63.2)	0.040
Cardiac disease, n (%)	6 (15.4)	6 (18.2)	3 (15.8)	0.958
Chronic respiratory disease, n (%)	2 (5.1)	3 (9.1)	3 (15.8)	0.403
Hypothyroidism, n (%)	2 (5.1)	2 (6.1)	1 (5.3)	0.984
COVID-19 status (positive within three months), n (%)	2 (5.1)	6 (18.2)	3 (15.8)	0.204
Past history of abdominal surgery, n (%)	26 (66.7)	14 (42.4)	9 (47.4)	0.099
Past history of bowel obstruction, n (%)	21 (53.8)	13 (39.4)	3 (15.8)	0.021
Pulse, BPM, mean ± SD	89 ± 15	90 ± 22	96 ± 24	0.576
Pyrexia (above 99 F), n (%)	2 (5.1)	2 (6.1)	5 (26.3)	0.026
Leukocyte count, 10^3^ per cu. mm, mean ± SD	11.6 ± 9.5	10.8 ± 4.1	13.6 ± 6.3	0.173
Neutrophil-to-lymphocyte ratio, mean ± SD	3.15 ± 0.44	3.29 ± 0.42	3.35 ± 0.73	0.031
Serum creatinine, mg/dL, mean ± SD	0.99 ± 0.39	0.97 ± 0.44	1.11 ± 0.65	0.749
Serum lactate at presentation, mg/dL, mean ± SD	14.06 ± 4.58	19.13 ± 7.20	32.70 ± 19.21	<0.001
Serum lactate after fluid resuscitation, mg/dL, mean ± SD	12.04 ± 2.96	21.98 ± 7.71	31.88 ± 13.90	<0.001
Level of obstruction (small bowel), n (%)	28 (71.8)	22 (66.7)	14 (73.7)	0.837
Adhesions, n (%)	20 (51.3)	11 (33.3)	5 (26.3)	0.124
Malignancy, n (%)	10 (25.6)	5 (15.2)	1 (5.3)	0.144
Obstructed hernia, n (%)	7 (17.9)	16 (48.5)	5 (26.3)	0.018
Inflammatory stricture, n (%)	2 (5.1)	3 (9.1)	1 (5.3)	0.769

**Figure 2:**
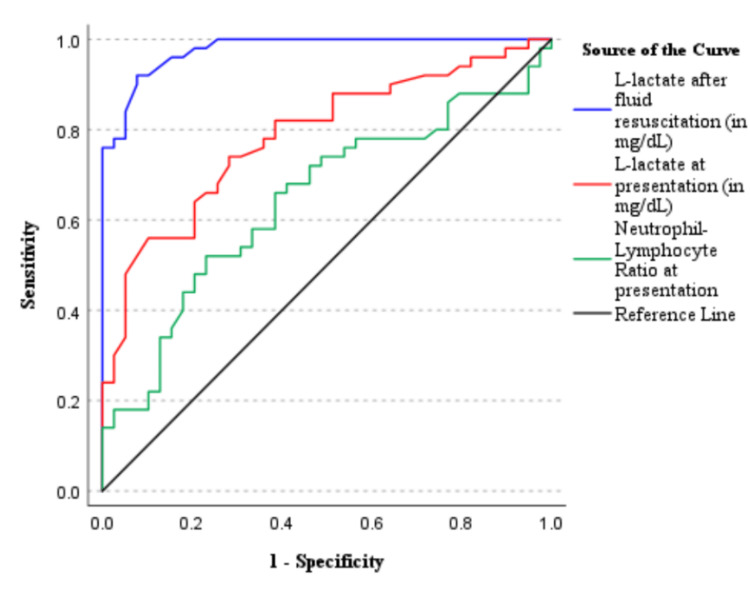
Receiver operating characteristic (ROC) curve for neutrophil-lymphocyte ratio and serum lactate values at presentation and after fluid resuscitation as predictors of intraoperatively observed bowel ischemia, reversible and irreversible (n = 52).

**Figure 3:**
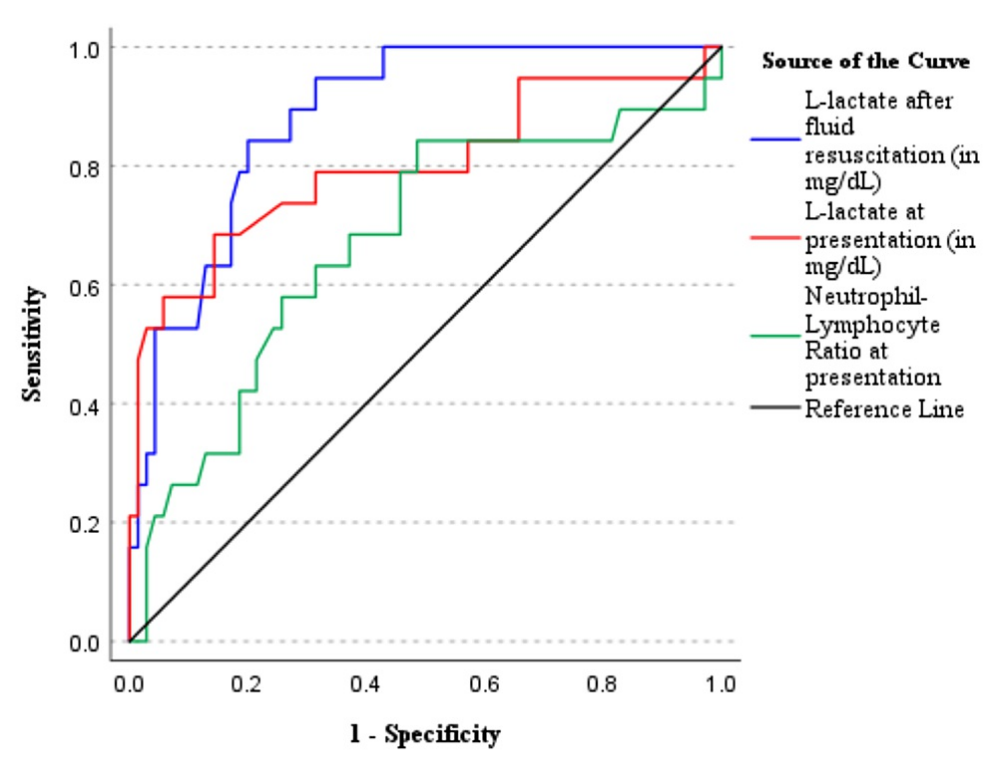
Receiver operating characteristic curve for neutrophil-lymphocyte ratio and serum lactate values at presentation and after fluid resuscitation as predictors of intraoperatively observed irreversible bowel ischemia (n = 19)

Patients were categorized twice for the purpose of ROC analysis: first, as having ischemic bowel (reversible and irreversible, n=52; Figure 2) vs. non-ischemic bowel, and second, as having irreversibly ischemic bowel (n=19; Figure 3) vs. salvageable bowel (non-ischemic or reversibly ischemic bowel). The predictive values of serum L-lactate at presentation and after fluid resuscitation, as well as the neutrophil-to-lymphocyte ratio, were compared in both categories.

On ROC curve analysis, the areas under the curve (AUC) for serum lactate at presentation and after fluid resuscitation for detection of bowel ischemia, both reversible and irreversible (n = 52), were 0.786 (95% confidence interval (CI), 0.692-0.880) and 0.977 (95% CI, 0.954-1.000; Figure 2), respectively. After resuscitation, an L-lactate threshold of 15.5 mg/dL yielded a sensitivity of 92.0%, a specificity of 92.3%, a positive predictive value of 94.1%, and a negative predictive value of 89.7% for predicting ischemia.

With respect to the detection of irreversible bowel ischemia (n = 19), the AUC of serum L-lactate at presentation and after fluid resuscitation were 0.797 (95% CI, 0.659-0.935) and 0.884 (95% CI, 0.812-0.956), respectively (Figure 3). A threshold of 19.1 mg/dL yielded a sensitivity of 73.7%, a specificity of 72.9%, a positive predictive value (PPV) of 43.1%, and a negative predictive value (NPV) of 91.4% at presentation, as opposed to a sensitivity of 89.5%, a specificity of 72.9%, a PPV of 46.6%, and an NPV of 96.3% after fluid resuscitation.

Serum L-lactate for predicting failure of conservative management

The demographic and clinical data of patients in whom non-operative management was attempted are represented in Table 2. Serum L-lactate after fluid resuscitation was significantly elevated in patients with failure of NOM (p < 0.001). The AUC for L-lactate after resuscitation and failure of NOM was 0.754 (95% CI, 0.624-0.885). A threshold of 15.5 mg/dL following adequate fluid resuscitation had a sensitivity of 52.2% and a specificity of 92.5%.

**Table 2:** Demographic variables, comorbidities, and clinical data in patients that underwent a trial of non-operative management (n = 76) Continuous variables: mean ± SD and Mann-Whitney U test results; categorical variables: n (%) and Pearson's chi-square test results; NA, not applicable

Variables	Non-operative management: successful (n=53)	Non-operative management: not successful (n=23)	p-value
Age, years, mean ± SD	56 ± 18.8	57 ± 18.8	0.726
Gender (male), n (%)	34 (64.2)	11 (47.8)	0.183
Diabetes, n (%)	15 (28.3)	5 (21.7)	0.551
Hypertension, n (%)	13 (24.5)	9 (39.1)	0.197
Cardiac disease, n (%)	10 (18.9)	2 (8.7)	0.264
Chronic respiratory disease, n (%)	4 (7.5)	2 (8.7)	0.865
Hypothyroidism, n (%)	8 (15.1)	1 (4.3)	0.183
COVID status (positive within three months), n (%)	7 (13.2)	3 (13.0)	0.984
Past history of abdominal surgery, n (%)	39 (73.6)	14 (60.9)	0.268
Past history of bowel obstruction, n (%)	41 (77.4)	13 (56.5)	0.066
Pulse, BPM, mean ± SD	90 ± 19	92 ± 17	0.606
Pyrexia (above 99 F). n (%)	5 (9.4)	1 (4.3)	0.450
Leukocyte count, 10^3^ per cu. mm, mean ± SD	10.87 ± 3.18	11.20 ± 4.31	0.384
Neutrophil-to-lymphocyte ratio, mean ± SD	3.22 ± 0.46	3.20 ± 0.42	0.756
Serum creatinine, mg/dL, mean ± SD	0.94 ± 0.29	1.02 ± 0.49	0.767
Serum lactate at presentation, mg/dL, mean ± SD	14.35 ± 5.80	15.31 ± 5.13	0.415
Serum lactate after fluid resuscitation, mg/dL, mean ± SD	10.87 ± 3.18	16.47 ± 8.24	<0.001
Level of obstruction (not small bowel), n (%)	NA	5 (21.7)	-
Ischemia of the bowel (reversible), n (%)	NA	9 (39.1)	-
Ischemia of the bowel (irreversible), n (%)	NA	0	-

## Discussion

Early and reliable diagnosis of intestinal ischemia is important, and the demand for easily accessible biomarkers remains high [8,9]. L-lactate assays based on the enzymatic reaction between L-lactate and either L-lactate oxidase or L-lactate dehydrogenase, both of which are specific for the substrate [11], are routinely available for point-of-care testing even in resource-poor settings [15]. This study aimed to evaluate serum L-lactate as a predictor of bowel ischemia in patients with intestinal obstruction.

On ROC analysis, serum L-lactate was found to be a good predictive factor of irreversible ischemia (AUC = 0.797, 95% CI, 0.659-0.935 vs. 0.884, 95% CI, 0.812-0.956, before and after resuscitation), as well as of ischemia, reversible or irreversible (AUC = 0.786, 95% CI, 0.692-0.886 vs. 0.977, 95% CI, 0.954-1.000, before and after resuscitation).

Yamamoto et al. reported similar findings, with sensitivity, specificity, positive predictive value, and negative predictive value (NPV) of lactate above 15 mg/dl of 93%, 90%, 68%, and 98%, respectively, in predicting strangulation in cases of bowel obstruction [10]. Kintu-Luwaga et al. also concluded lactate was predictive of bowel ischemia in cases of mechanical bowel obstruction, with an NPV of 93% for irreversible ischemia [16]. Nuzzo et al. [17] reported serum lactate levels >2 mmol/l to be significantly associated with bowel necrosis on multivariate analysis (hazard ratio: 4.1 (95% CI: 1.4-11.5); p=0.01) for prediction of irreversible ischemia in cases of mesenteric ischemia.

Drawbacks reported in the current literature for the utility of serum L-lactate include its limitations in the diagnosis of acute mesenteric ischemia [18]. Matsumoto et al. reported an AUC of 0.72 (95% CI 0.58-0.86) for lactate in detecting vascular intestinal ischemia [19], while a review by Evenett et al. claimed a sensitivity ranging between 0.77 and 0.33 and specificity between 0.74 and 0.53 for L-lactate [8]. This low predictive value is especially notable in case series of patients with other underlying or predisposing conditions for elevated lactate, such as ICU patients in sepsis, post-cardiovascular surgeries, cases of acute abdomen under evaluation [18-20], etc. Additionally, in cases of short-segment ischemia, serum lactate may be unreliable in identifying bowel hypoperfusion [21]; studies have also failed to establish a linear relationship between serum lactate and the extent of bowel ischemia [22, 23].

This study sought to overcome the limitation of non-specificity by evaluating L-lactate as a predictive factor for bowel ischemia in patients already established to have acute intestinal obstruction. Common causes of lactic acidosis and potential confounding conditions, such as end-stage renal disease and diabetic ketoacidosis, were added as exclusion criteria. Cases detected to have pneumoperitoneum suggestive of hollow viscus perforation, as well as patients presenting in the immediate postoperative period, were excluded to avoid elevation of L-lactate secondary to sepsis, possible anastomotic leaks, or deep-space surgical site infections.

Since patients may present with lactic acidosis in intestinal obstruction due to dehydration, decreased oral intake, hypovolemia contributing to splanchnic hypoperfusion, etc. [14, 24], it was also attempted to determine whether fluid resuscitation improved the accuracy of serum lactate in identifying bowel ischemia. This study found that preoperative fluid resuscitation improved the predictive value of serum lactate in identifying both reversible and irreversible ischemia, which was consistent with Studer et al.’s reports of a decrease in lactate values following fluid resuscitation [22].

However, it could not be reliably determined whether this improved accuracy is due to the correction of dehydration as a confounding factor or due to progressive ongoing ischemic insult during the period of resuscitation. As per Cox et al., it is possible that by the time elevation of lactate develops to a degree sufficient to allow preoperative recognition of the ischemic process, the process may already be at an irreversible stage [25].

A significant limitation of this study is the lack of homogeneity in etiology and level of bowel obstruction. Cosse et al. reported no significant differences in lactate levels between patients with and without intraoperatively demonstrated ischemia in adhesive small bowel obstruction [26]. Additionally, though the elimination of potential confounding factors in lactate metabolism was attempted through study exclusion criteria, the possibility of lactate elevation secondary to previously unrecognized renal or hepatic failure or remote septic foci acts as a limitation of the study.

Several reasons have been proposed as explanations for the difficulty of finding a reliable early biomarker for intestinal ischemia [9,11]. In addition to the non-specificity of classical plasma biomarkers, the use of specific intestinal proteins may be limited by delayed release into circulation until the onset of severe mucosal damage or elimination by first-pass hepatic metabolism, preventing them from entering the systemic circulation. Alternatives such as intraoperative assessment of bowel viability using local capillary lactate assays and fluorescence-based laparoscopic augmented reality systems are under study [21,27,28].

Nevertheless, defining accuracy and sensitivity for intestinal ischemia with respect to easily available conventional systemic biomarkers like L-lactate may potentially help direct surgical therapy and support early intervention during the stage of reversible ischemia.

## Conclusions

This study evaluating the utility of serum L-lactate in the management of intestinal obstruction showed L-lactate was a good predictive biomarker of intestinal ischemia on ROC analysis. At a cut-off of 19.1 mg/dL, L-lactate was determined to have a sensitivity of 73.7% and an NPV of 91.4% at presentation, and a sensitivity of 89.5% and an NPV of 96.3% following fluid resuscitation for detecting irreversibly ischemic bowel.

Comparison of the predictive value of L-lactate before and after resuscitation using ROC analysis showed that the latter was more accurate for ischemia, reversible or irreversible (AUC = 0.786, 95% CI, 0.692-0.886 vs. 0.977, 95% CI, 0.954-1.000), as well as for irreversible ischemia (AUC = 0.797, 95% CI, 0.659-0.935 vs. 0.884, 95% CI, 0.812-0.956). Additionally, L-lactate after fluid resuscitation has a fair predictive value of failure of non-operative management in adhesive small bowel obstruction with an AUC of 0.754.

Serum L-lactate is an easily accessible biomarker with good sensitivity for intestinal ischemia in cases of bowel obstruction and can be reliably used to anticipate ischemic changes in the bowel. This will help plan surgical intervention and anticipate the termination of non-operative management in cases of adhesive small bowel obstruction. Due to the lack of homogeneity of etiology and level of obstruction in this study population, further studies are required to support this conclusion.
